# Body weight in midlife and long-term risk of developing heart failure-a 35-year follow-up of the primary prevention study in Gothenburg, Sweden

**DOI:** 10.1186/s12872-015-0008-2

**Published:** 2015-03-10

**Authors:** Lena Björck, Masuma Novak, Maria Schaufelberger, Kok Wai Giang, Annika Rosengren

**Affiliations:** Department of Molecular and Clinical Medicine, University of Gothenburg, Gothenburg, Sweden; Institute of Health and Care Sciences, Sahlgrenska Academy, University of Gothenburg, Gothenburg, Sweden

**Keywords:** Epidemiology, Heart failure, Midlife, Obesity, Overweight, Long-term risk

## Abstract

**Background:**

This study aimed to determine whether midlife obesity predicts heart failure (HF) over an extended follow-up into old age.

**Methods:**

We studied 7495 men (from a population sample of 9,998 men) without HF, who were 47–55 years old when investigated in 1970 to 1973. All participants were followed up for 35 years, or until death, using the Swedish National Inpatient Register (IPR) and the Cause of Death Register. Over follow-up, 1855 men (24.7%) were discharged from hospital or died with a diagnosis of HF.

**Results:**

There was a strong relation between obesity and future risk of HF, which was accentuated over the last years of the long follow-up. After adjusting for age, the risk of HF increased stepwise with increasing body mass index (BMI), even in those with a normal BMI (22.5–24.9) The subdistribution hazard ratio (SHR) was 1.20 (95% CI: 1.02–1.39) in men with a normal BMI, 1.29 (95% CI: 1.11–1.50) for a BMI of 25–27.49, 1.50 (95% CI: 1.27–1.77) for a BMI of 27.5–29.99, and 1.62 (95% CI: 1.33–1.97) for a BMI >30. After adjusting for, age, smoking, occupational class, and physical activity, the results were unchanged.

**Conclusion:**

Obesity in midlife is strongly related to the long-term risk of developing HF extending into old age where the risk is highest. Even normal body weight (BMI <25) was related to an increased risk of developing HF during life. Because overweight and obesity are largely preventable, our findings further emphasize the importance of public health interventions against the development of obesity.

## Background

Overweight and obesity is an increasing problem worldwide. Since 1980, obesity has doubled worldwide with an even larger increase in some countries [1]. A number of diseases, such as diabetes, cancer, dementia, and cardiovascular disease, including heart failure (HF) are related to overweight and obesity [2,3]. The link between obesity and HF is well established, and obesity is now recognised as a major risk factor for developing HF in the general population, including elderly people [4-6].

HF is a common disease with an estimated prevalence of approximately 2–2.5% in Sweden and other Western countries and is more common in men than women [7-11]. The prevalence of HF is strongly related to age, increasing steeply from <1% in those aged 19 to 54 years, to about 10% in age 85 and 20% in ages above 85 [11]. Even if the incidence of HF is decreasing [7,11,12], which is the case in Sweden, an increasingly larger proportion of elderly people in the population means that HF will be a rising problem. Additionally, this increase could be accentuated by an increasing proportion of overweight and obese persons.

Even though the relation between obesity and HF is well known, there is still a lack of information on to which extent midlife obesity can predict HF in old age, where the risk is highest. Further, the level of body weight at which risk of HF starts to rise has not been determined. Therefore, using data from a cohort of middle aged men from a comparatively narrow age range who were followed up for 35 years or until they died, we aimed to investigate the association between different levels of body weight and the cumulative risk of being hospitalized with HF.

## Methods

Data were derived from male participants from the intervention group in the multifactor Primary Prevention Study, which began in Gothenburg, Sweden, in 1970 [13]. All men in the city born between 1915–1925 (n = 30,000), except those born in 1923 (because they were part of another study), were randomized into three groups of 10,000 men each (one intervention group and two control groups). The men in one of the groups (intervention group; n = 9,998) were invited to a screening examination to identify and treat risk factors. The intervention was essentially a high-risk strategy directed towards men with pronounced hypercholesterolaemia, severe hypertension, or heavy smoking habits, according to pre-defined criteria, with treatment offered at specialist clinics. The first screening examination included 7,495 men (non-participation rate of 25% (2,503 men) in the intervention group and took place between January 1970 and March 1973. All men were free from known HF at the time of the first screening. Limited subsamples of the control groups were examined but apart from that no baseline data were collected in these groups. During the first 12-year follow-up, there were no significant differences in outcome with respect to cardiovascular disease, cancer, or all-cause mortality between the intervention group and the two control groups [1,13]. Therefore, despite the fact that the men took part in an intervention study, we consider the study group to be representative of the general Gothenburg male population. All participants gave their informed consent to participate in the study. The study was approved by the Ethics Committee for Medical Research at the University of Gothenburg.

Information on risk factors (smoking, physical activity during leisure time, occupation, and diabetes) were obtained by questionnaire. Screening examinations were performed in the afternoon. Weight was measured in kilograms to the nearest 0.10 kg and height was measured in meters to the nearest 0.01 m. BMI was calculated as weight in kilograms divided by the square of height in meters. We used five categories of BMI: <22.5, 22.5-24.9, 25–27.49, 27.5-29.9, and ≥30 kg/m^2^, corresponding to low normal, high normal, slightly overweight, overweight and obese. Blood pressure (BP) was measured to the nearest 2 mm Hg in the sitting position after 5 min of rest. Hypertension was defined as systolic blood pressure (SBP) >140 mm Hg, diastolic blood pressure (DBP) >90 mm Hg, or taking antihypertensive treatment. Total serum cholesterol levels were measured after fasting for ≥2 h and were determined according to standard laboratory procedures. Smoking habits and a previous history of ischemic heart disease (IHD) were obtained from the baseline questionnaire. Smoking was defined using two categories: current smoker or non-smoker (never or former smoker [>1 month]). Physical activity during leisure time was categorized into three levels: sedentary, moderate activity at least 4 hours per week, and regular, strenuous activity for at least 2–3 h per week. Occupation was used to classify men into five occupational classes and was coded according to the Swedish socio-economic classification system, the Socio-Economic Index (SEI): (1) unskilled and semiskilled workers, (2) skilled workers, (3) foremen in industrial production and assistant non-manual employees, (4) intermediate non-manual employees, and (5) employed and self-employed professionals, higher civil servants, and executives. In this study, we divided occupational status into three groups using the SEI classification system: low (SEI codes 1, 2), intermediate (SEI codes 3, 4), and high occupational status (SEI code 5).

### Follow-up

All participants in the study were followed up from their baseline examination until December 31, 2008, using the personal identification number unique to all Swedish citizens. A file with all participants was run against the Swedish national Inpatient Register (IPR) and the Swedish Cause of Death register. The IPR, established in 1964, has operated on a nationwide basis since 1987, but all discharges from Gothenburg hospitals have been entered in the national register since 1970 (except for 1976 owing to a legislative change for that single year) [14]. Loss due to emigration was negligible. HF was defined as discharge with a primary or secondary diagnosis of HF using the Swedish *International Classification of Disease* (ICD) code of 427.00 or 427.10 for ICD-8, 428A, 428B, or 428X for ICD-9, or I50 for ICD-10.

For the purpose of identifying HF cases due to IHD, we categorized all cases with a discharge diagnosis of non-fatal acute myocardial infarction (AMI) and/or coronary revascularization, either before or at any time after diagnosis of HF due to IHD. Non-fatal myocardial infarction was defined as a discharge code of 410 (ICD-8 and −9) or I21 (ICD-10). Coronary revascularization was defined as any discharge with a discharge code of 410–414 (ICD-8 and −9) or I20-I25 (ICD-10) and an operation code of any of 3066, 3080, 3067, 3127, 3091, 3029, FNA, FNC, or FNG.

### Statistical analysis

All statistical analyses were performed using SAS software version 9.2 (SAS Institute Inc, Cary, North Carolina) and R statistical system (version 2.15.1). Descriptive statistics with baseline characteristics are presented for each BMI class and risk factors. A major problem for long-term follow up studies is the presence of competing events, such as death. This could potentially end the follow-up for a study subject in such a way that violates the random censoring assumption when calculating risk differences in risk factors. In the present study, a competing risk regression was used to study the long-term risk of HF by risk factors and BMI group. For this purpose, a modified Cox proportional hazard regression analysis was used, yielding a subdistribution hazard ratio (SHR) with two sided 95% confidence intervals (CIs) [2,3,15,16]. All estimates for the risk of developing HF by BMI groups were adjusted for age, IHD, smoking, physical activity, total serum cholesterol levels, diabetes, hypertension, and occupational class where each categorized risk level (based on the different levels of the categorized risk factors) was compared with the corresponding reference group. In addition, a cumulative incidence curve was estimated. The R package “cmprsk”, which is publicity available at the R archive network site (http://cran.r-project.org/), was used to calculate the SHR and cumulative incidence.

## Results

Of the 7,495 men with no HF at time of the baseline examination, 57 had prior self-reported IHD. The total mean BMI was 25.5 (standard deviation [SD] ±3.1/kg/m^2)^. The overall mean age was 51.1 years, with no difference between the BMI groups. As expected, serum cholesterol, diabetes, SBP, and hypertension increased with rising BMI (Table 1). Smoking was most common in those with a BMI <22.5 (65.1%) and lowest in those with a BMI ≥30 (44.2%). BMI relative to occupational class showed an inverted J-shaped curve with a higher incidence of overweight and obesity in individuals with intermediate occupational class compared with those with high and low occupational class (Table 1).

**Table 1:** **Baseline characteristics in 7,495 men by body mass index (BMI) category**

Number of men*	**All**	**BMI 22.5**	**BMI 22.5-24.9**	**BMI 25–27.49**	**BMI 27.5-29.9**	**BMI(≥30)**
	**(n = 7495)**	**(n = 1180)**	**(n = 2267)**	**(n = 2222)**	**(n = 1207)**	**(n = 617)**
Age, years, mean (SD)	51.1 (2.3)	51.0 (2.3)	51.1 (2.3)	51.2 (2.3)	51.2 (2.4)	51.1 (2.3)
S-cholesterol mmol/L, mean (SD)	6.46 (1.16)	6.19 (1.10)	6.37 (1.15)	6.53 (1.15)	6.67 (1.13)	6.65 (1.22)
SBP mm Hg, mean (SD)	149 (22)	143 (21)	146 (21)	149 (22)	154 (22)	159 (22)
Hypertension, % (n)	70. 1 (5245)	58.1 (685)	64.9 (1467)	71.9 (1595)	79.9 (963)	86.7 (535)
Diabetes, % (n)	2.0 (149)	2.7 (32)	1.5 (35)	1.7 (38)	2.0 (24)	3.2 (20)
IHD, % (n)	57 (0.76)	10 (0.85)	10 (0.44)	18 (0.81)	13 (1.08)	6 (0.97)
Current smoker	50.3 (3768)	65.1 (769)	52.9 (1199)	45.2 (1005)	44.2 (533)	42.5 (262)
Physical activity, % (n)						
Regularly	15.8 (1166)	15.2 (176)	17.0 (383)	17.9 (393)	12.0 (144)	11.7 (70)
Moderate	58.3 (4308)	57.5 (664)	60.1 (1360)	58.0 (1272)	58.1 (695)	52.8 (317)
Inactive	26.0 (1920)	27.3 (315)	22.5 (505)	24.1 (529)	29.9 (357)	35.6 (214)
Occupational class						
High	27.7 (2078)	26.8 (317)	29.9 (678)	28.7 (637)	24.7 (298)	24.0 (148)
Intermediate	44.2 (3314)	44.3 (524)	44.0 (997)	45.2 (1005)	44.1 (532)	41.5 (256)
Low	28.1 (2102)	28.9 (341)	26.1 (591)	26.1 (580)	31.2 (377)	34.5 (213)

*If numbers do not add up to the total number it is due to missing data.

BMI indicates body mass index; SBP systolic blood pressure; and IHD ischemic heart disease.

Study participants were followed for 189 934 person-years during the 35-year follow-up, during which 1855 men (24.7%) were hospitalized with a diagnosis of HF. Men with a BMI <22.5 had the lowest risk of HF and were used as the reference group. After adjusting for age, the risk of HF increased stepwise with increasing BMI, with a discernible increase in risk even in men with a normal BMI of 22.5–24.99 [SHR: 1.20, 95% CI: 1.02–1.39]. The SHR was 1.29 (95% CI: 1.11–1.50) in men with a BMI of 25–27.49, 1.50 (95% CI: 1.27–1.77) in those with a BMI of 27.5–29.99, and 1.62 (95% CI: 1.33–1.97) in those with a BMI >30 (Table 2). After adjusting for age, IHD, smoking, occupational class, and physical activity, the result were only slightly changed (Table 2).

**Table 2:** **Lifetime risk of developing heart failure in 7,495 men by body mass index (BMI) with or without ischemic heart disease**

	**Number at risk**	**HF events**	**Observation years**	**Incidence rate per 1000 observations years**	**Age adjusted SHR**	**Multivariable adjusted** ^**a**^ **SHR**	**Multivariable adjusted** ^**b**^ **SHR**
	**n = 7494**				**(95% CI)**	**(95% CI)**	**(95% CI)**
**All HF events (n = 1855)**							
**BMI**							
<22.5	1182	234	29473	7.94	1.00 (ref)	1.00 (ref)	1.00 (ref)
22.5-24.99	2266	533	59057	9.03	1.20 (1.02-1.39)	1.19 (1.02-1.39)	1.16 (0.99-1.36)
25-27.49	2222	562	57564	9.76	1.29 (1.11-1.50)	1.30 (1.11-1.52)	1.26 (1.07-1.47)
27.5-29.9	1207	342	29864	11.45	1.50 (1.27-1.77)	1.51 (1.28-1.79)	1.44 (1.21-1.71)
≥30	617	184	13976	13.17	1.62 (1.33-1.97)	1.61 (1.32-1.96)	1.51 (1.23-1.85)
**HF without IHD (n = 851)**							
**BMI**							
<22.5	1182	117	29888	3.91	1.00 (ref)	1.00 (ref)	1.00 (ref)
22.5-24.99	2266	251	60084	4.18	1.11 (0.89-1.38)	1.08 (0.87-1.35)	1.11 (0.88-1.38)
25-27.49	2222	247	58580	4.22	1.11 (0.89-1.38)	1.10 (0.89-1.37)	1.14 (0.91-1.43)
27.5-29.9	1207	153	30603	5.00	1.28 (1.01-1.63)	1.28 (1.00-1.63)	1.38 (1.07-1.77)
≥30	617	83	14352	5.78	1.39 (1.05-1.84)	1.39 (1.04-1.85)	1.52 (1.13-2.04)
**HF and IHD (n = 1004)**							
**BMI**							
<22.5	1182	117	29785	3.93	1.00 (ref)	1.00 (ref)	1.00 (ref)
22.5-24.9	2266	282	59844	4.71	1.26 (1.02-1.57)	1.28 (1.02-1.58)	1.20 (0.97-1.50)
25-27.49	2222	315	58386	5.40	1.44 (1.17-1.79)	1.47 (1.18-1.83)	1.34 (1.07-1.67)
27.5-29.9	1207	189	30440	6.21	1.62 (1.29-2.04)	1.65 (1.30-2.08)	1.43 (1.12-1.81)
≥30	617	101	14354	7.04	1.72 (1.32-2.25)	1.69 (1.29-2.23)	1.41 (1.07-1.86)

^a^Adjusted for age, IHD, smoking, physical activity and occupational status.

^b^Adjusted for age, IHD, smoking, physical activity, occupational class, total serum cholesterol, diabetes, systolic blood pressure and hypertension.

^c^IHD defined as acute myocardial infarction or revascularization (coronary artery bypass grafting (CABG) or percutaneus coronary intervention (PCI)).

HF indicates heart failure; BMI, body mass index; SHR, subdistribution hazard ratio and IHD, ischemic heart disease.

After further adjustment for total s-cholesterol, SBP, hypertension, and diabetes, which are strongly associated with body weight and may be regarded as mediators in the causal chain, there was no significant increase in risk in men with a BMI of 22.5–24.9 (SHR: 1.16; 95% CI: 0.99–1.36), while the risk was still increased among overweight men (BMI of 25–27.49: SHR, 1.26 [95% CI: 1.07–1.47]; BMI of 27.5–29.9: SHR, 1.44 [95% CI: 1.21–1.71]; BMI ≥30: SHR, 1.51 [95% CI: 1.23-1.85]) (Table 2).

The study population was then divided into HF without overt IHD (45.9% of all HF) and HF with IHD in relation to the BMI group. IHD was defined as prior AMI or cardiovascular intervention (percutaneous coronary intervention or coronary artery bypass grafting) at any time prior or post HF. However, there was no evident difference in the relation between midlife BMI and risk of HF between the groups with and without IHD (Table 2).

**Figure 1:**
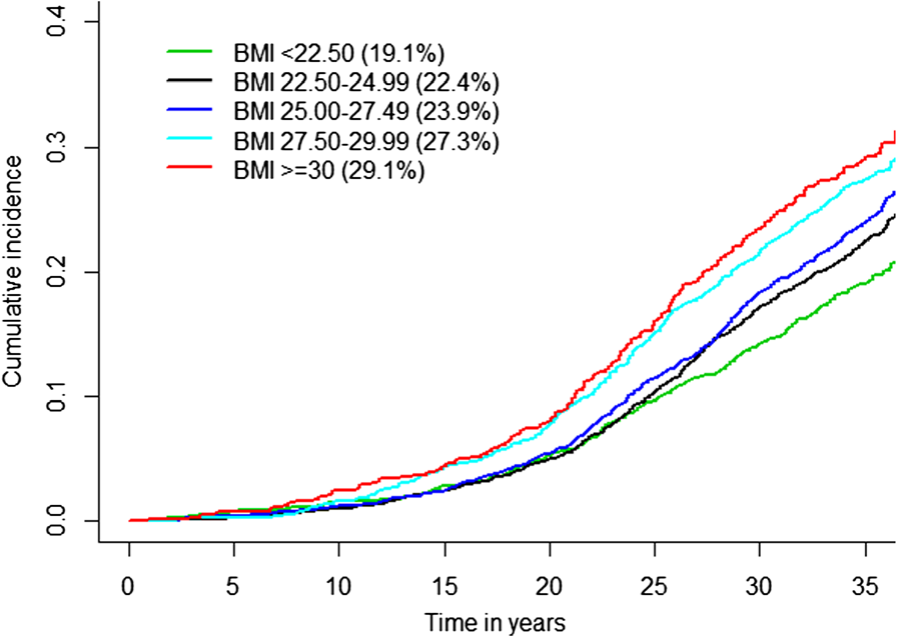
**Cumulative incidence and long-term risk of developing heart failure by BMI group.**

In addition, we calculated the cumulative incidence for HF, adjusted for competing risk, and further showed an increasing incidence of HF over life in relation to the BMI group. The strong relation between overweight and obesity in midlife and the future risk of HF was accentuated over the last years of the long follow-up, with more than half of the cases occurring during the last 10 years of the study (Figure 1). A marked increase in hospitalization for HF was observed 15 to 20 years after the baseline examination.

Even in men with a BMI <25 in midlife, which is considered as normal weight, the cumulative risk of HF was between 19.1% and 22.4%, increasing progressively to 23.9% and 27.3% in men with a BMI ≥25 to <30 and to 29.1% in those with a BMI ≥30.

## Discussion

In the present study, we found that weight in midlife was strongly related to the future risk of HF, extending into old age where the majority of cases occur. Even weight considered as normal (BMI of 22.5–25), not only overweight or obesity, was related to an increased risk of developing HF. This finding suggests a low threshold with respect to body weight and the risk of developing HF. Adjustment for other risk factors did not substantially alter our results. However, since symptoms in HF and in obesity partly are similar HF could be detected more often in persons with higher BMI. It has earlier been shown that the prevalence of subjective symptoms of HF in people with obesity is higher or similar but that the prevalence of objective signs is lower. Consequently patients with higher BMI could have been diagnosed at an earlier phase of HF than those with lower BMI which furthermore could have influenced the prevalence of HF [17].

HF increases steeply with age and is common in old age where the risk of mortality is high. The cumulative risk method that we used assumes that people who die during the long follow-up period would have the same risk of developing the disease as those who survived. Therefore, we adjusted for competing risk to avoid overestimation of the long-term risk of HF. Even with this adjustment, final risk of HF after 35 years was substantial, adding to the literature that shows that HF is highly prevalent among elderly people [7,18]. A recent study, including all Swedish men and women hospitalized between 1990 and 2007 with a discharge diagnosis of HF, showed a prevalence of 9% at 75–84 years and a prevalence of 20% in those aged >85 years [11]. This is consistent with our findings where half of the HF cases occurred during the last 10 years of the follow-up when the men were aged between 80 and 85 years old.

HF is a major cause of morbidity and mortality in the population, not only at the community level, but also for individuals. Our study indicated the size of the problem and also suggested that HF will be a growing health issue in the future with the aging of the population. Moreover, with and an increasing prevalence of overweight and obesity in the population these results are highly relevant [19]. In addition, the total cost for HF patients, including hospital care, primary health care, nursing homes, investigations, and medication is high, and will increase further with an ageing population [20].

Potential mechanisms for the link between overweight/obesity include cardiac remodelling with left ventricular hypertrophy and left atrial enlargement. These are associated with diastolic dysfunction and its progression to systolic dysfunction, probably related to neurohormonal activation and oxidative stress [21-23] IHD is estimated to cause approximately half of all HF cases [24], and because obesity, especially abdominal obesity [25,26], is a risk factor for myocardial infarction, part of the increase in risk could be mediated by IHD. Nevertheless, we found that BMI in midlife was related to the risk of developing HF also in cases without IHD.

### Strengths and limitations

Our study showed that 24.7% of the men developed HF during the 35-years follow-up which is line with a recent Swedish study where the prevalence of HF in men aged >80 was 21.6% in age 89–89 and 30.3% in ages >90 [7]. The strength of our study is that we had a large sample size, with very long follow-up of over more than 3 decades with negligible loss of follow-up and providing a sufficient number of HF cases occurring in advanced age. However, there are also some limitations to our study. First, HF was defined as discharge with a primary or secondary diagnosis using the Swedish IPR registers, with data collected for administrative and not for research purposes. Even so, a hospital diagnosis of HF in Sweden in the relevant period has been shown to have good validity [27]. Secondly, HF was not assessed at base-line and men with prior HF could accordingly have been included in the study. However, since HF is rare in this age group this is unlikely to have had an effect [11]. Third, only those men who were hospitalized for HF were included in the study. This could have led to an underestimation of the incident cases in our study because milder cases of HF are handled in outpatient care [7]. Regardless, hospitalizations are considered to bear the brunt of cost and suffering in connection with HF. In addition, the study was designed as an intervention trial. However, no significant difference in outcome with respect to ischemic heart disease, cancer, or all-cause mortality was found between the intervention group and the two control groups [16].

In addition, our study included only men, which is a limitation because HF is common in men and women. Accordingly the conclusions of the study are limited to men. Also, we only had information on BMI at baseline and at the re-examination four years after the baseline examination and no information on body weight or weight change later in life, which may also influence the life risk of developing HF. Misclassification of any given exposure may obviously influence results. However, our results indicate that overweight and obesity in midlife, without further information on subsequent weight development, is a strong predictor for HF in advanced age.

## Conclusions

In our sample of men, the long-term risk of being hospitalized with HF was considerable, and increased incrementally with midlife body weight at all levels beyond a BMI of 22.5 kg/m^2^. This was partly mediated by the effect of body weight on cholesterol, diabetes, SBP and hypertension such that there was overall no independent effect below a BMI of 25 kg/m^2^. Still, the study shows that even moderately increased body weight in midlife, not only obesity, can predict the risk of developing HF later in life. Our findings further emphasize the importance of public health measures in order to prevent the development overweight and obesity in middle-aged people.
